# Sodium Hyaluronate Supplemented Culture Media as a New hMSC Chondrogenic Differentiation Media-Model for *in vitro/ex vivo* Screening of Potential Cartilage Repair Therapies

**DOI:** 10.3389/fbioe.2020.00243

**Published:** 2020-03-31

**Authors:** Graziana Monaco, Alicia Jennifer El Haj, Mauro Alini, Martin James Stoddart

**Affiliations:** ^1^AO Research Institute Davos, Davos, Switzerland; ^2^School of Pharmacy and Bioengineering, Faculty of Medicine and Health Sciences, Keele University, Guy Hilton Research Centre, Thornburrow Drive, Stoke-on-Trent, United Kingdom; ^3^Healthcare Technology Institute, Institute of Translational Medicine, University of Birmingham, Birmingham, United Kingdom

**Keywords:** hyaluronic acid, mesenchymal stem cells, chondrogenic differentiation media, glycosaminoglycan, collagen X, hypertrophy, articular cartilage, synovial fluid

## Abstract

Surgical strategies to treat articular cartilage injury such as microfracture, expose human bone marrow stem cells (hMSCs) to synovial fluid and its components. High molecular weight hyaluronan (hMwt HA) is one of the most abundant bioactive macromolecules of healthy synovial fluid (hSF) and it plays an important role in the protection of opposing articular cartilage surfaces within the synovial joint. Although hMwt HA has been extensively used to attempt the engineering of the cartilage tissue, its effect as media supplement has not been established. Indeed, current media are often simple in their composition and doesn’t recapitulate the rheological and biological features of hSF. In addition, critical *in vivo* molecules that can potentially change the chondrogenic behavior of hBMSCs to make the *in vitro* results more predictive of the real *in vivo* outcome, are lacking. In order to be one step closer to the *in vivo* physiology of hSF, a new culture media supplemented with physiological level of hMwt HA was developed and the effect of the hMwt HA on the chondrogenesis of hMSCs that would be present in a traumatic defect after marrow stimulation techniques, was investigated. hBMSC-seeded fibrin-polyurethane constructs were cultured in a serum free chondropermissive control medium (HA- TGFβ-). This medium was further supplemented with 10 ng/mL TGFβ1 (HA- TGFβ+) or 2 mg/ml hMwt HA 1.8 MDa (HA+ TGFβ-) or both (HA+ TGFβ+). Alternatively, 1 MDa HA was mixed with the fibrin at 0.2 mg/ml (HASc TGFβ+). The effect of hMwt HA on hMSC differentiation was investigated at the gene expression level by RT-qPCR and total DNA, sulfated glycosaminoglycans and Safranin O staining were evaluated. Addition of hMwt HA to the culture media, significantly increased the synthesis of sulfated glycosaminoglycans, especially in the early days of chondrogenesis, and reduced the upregulation of the hypertrophic cartilage marker collagen X. hMwt HA added inside the fibrin gel(HASc TGF+) led to the best matrix deposition. hMwt HA can be one key medium component in a more reliable *in vitro/ex vivo* system to reduce *in vitro* artifacts, enable more accurate pre-screening of potential cartilage repair therapies and reduce the need for animal studies.

## Graphical Abstract

Bioengineered media model for hMSCs chondrogenic differentiation.

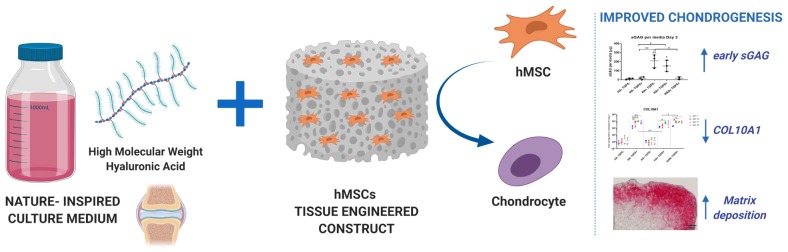

## Introduction

Articular cartilage is a complex anisotropic tissue which consists of a superficial zone, middle zone, deep zone, and calcified zone. Each zone has a well-defined structure with characteristic collagen fiber organization and is mainly populated by the chondrocyte, which is responsible for extracellular matrix maintenance (Klein et al., 2009; Stoddart et al., 2009).

Articular cartilage covers the osseous ends of articulating diarthrosis also known as synovial joints and in this anatomical context is in contact with Synovial fluid.

The synovial fluid (SF) of healthy joints normally functions as a biological lubricant as well as a biochemical depot through which nutrients and regulatory cytokines are transported.

Synovial fluid which is derived from the ultrafiltration of the blood plasma and glycoproteins, is concentrated inside the joint by passing through the synovium, a fibrous, highly vascularized membrane whose internal layer macrophage-like synovial cells (type A) and type B synoviocytes resides.

High molecular weight hyaluronan (HA), is one of the major components of healthy SF that undergoes dynamic regulation during cartilage damage and inflammation. It plays an important role in the protection of opposing articular cartilage surfaces by improving joint lubrication, but is also involved in nutrient transport to the articular cartilage tissue, it has an excellent osmotic buffering property to maintain water homeostasis inside the joint, acts as pressure regulator and thanks to its gel-like structure, prevents tissue formation in the synovial space (McDonald and Levick, 1995; Laurent et al., 1996; Lynch et al., 1998).

Hyaluronan is composed of a repetitive sequence disaccharide unit which consist of 1,3-beta-D-N-acetylglucosamine and 1,4-beta-D-glucuronic acid and possess particular features that makes it a unique biopolymer (Jiang et al., 2006). It is produced by specific multipass transmembrane enzymes, the HA synthases 1–3, located on the inner surfaces of the plasma membranes (Weigel et al., 1997; Itano and Kimata, 2002; Kogan et al., 2007) and is continuously secreted into the joint and cleared with a half-life of 0.5–1 day in rabbit or sheep (Brown and Laurent, 1991; Fraser et al., 1993).

In healthy synovial fluid high molecular weight hyaluronan can achieve a molecular mass of 6–10 mega Dalton (>10^6^ Dalton) but when inflammation or oxidative stress occurs, an accelerated HA degradation into smaller fragments has been observed (Noble, 2002; Jiang et al., 2011; Fakhari and Berkland, 2013). As such, the molecular weight of HA inside the SF is representative of the physio-pathological condition of the tissue. High molecular weight HA (10^5^–10^7^ Da) is protective for synovial joint and articular cartilage showing anti-inflammatory, anti-angiogenic properties, stimulation of chondrocyte proliferation and production of cartilage matrix and scavenging function against cellular debris and oxidizing systems (Laurent et al., 1996; Gigante and Callegari, 2011; Gallo et al., 2019). Conversely, low molecular weight HA is pro-inflammatory, pro-angiogenic and immune-stimulatory reflecting a tissue under stress as happen in rheumatoid/inflamed joints (Jiang et al., 2011; Gallo et al., 2019).

Due to its avascular nature, partial thickness defects or disease of articular cartilage cannot be easily accessed by stem cells. This condition associated with low metabolic activity, make unsuccessful self-healing and repair (Hunziker, 2000; Huey et al., 2012).

Microfracture treatment of articular cartilage injury is the standard clinical practice to facilitate the access of mesenchymal stem cells (MSCs) that reside in the bone marrow cavity of the subchondral bone at the injured site, promoting cartilage regeneration (Steadman et al., 2001; Kang et al., 2008; Oussedik et al., 2015). These cells would be exposed to synovial fluid and the HA contained within.

Human mesenchymal stem cells derived from bone marrow (hBM MSCs) represent an attractive cell source for cartilage tissue engineering and regenerative medicines approaches, are the best described, the most advanced in clinical use and can differentiate in cartilage or bone (Wakitani et al., 2002; Parekkadan and Milwid, 2010; de Vries-van Melle et al., 2014). hBM MSCs can be easily harvested and isolated from bone marrow aspirates with limited donor site morbidity; in addition, following expansion BM MSCs maintain multilineage potential (Hegewald et al., 2004; Gardner et al., 2013). It has been shown that MSCs are able to repair both the subchondral bone and the overlying articular cartilage, and many studies describe how MSCs are useful in cartilage repair after injury or disease such as osteoarthritis (Wakitani et al., 1994; Wakitani et al., 2002).

One of the main problems associated with the chondrogenic differentiation of hBM MSCS *in vitro*, is that MSC-derived chondrocytes undergo hypertrophic differentiation that causes the neo-formed cartilage to undergo endochondral ossification (Johnstone et al., 1998).

HA has been extensively used as a polymer in scaffolds or hydrogel form, alone or in combination with MSCs to produce engineered cartilage with native tissue properties (Radice et al., 2000; Solchaga et al., 2000; Huerta-Ángeles et al., 2018; Li et al., 2018; Gallo et al., 2019).

Although it represents one of the most abundant macromolecules of the synovial fluid, it has not been extensively studied as media supplement to evaluate its effect on chondrogenesis.

Indeed, culture media currently used to induce chondrogenic differentiation of hMSCs are often simple in their composition and doesn’t recapitulate the rheological and biological features of knee joint synovial fluid (Heng et al., 2004).

In this study, we hypothesize that a chondropermissive medium containing a physiological concentration (2 mg/ml) of exogenous high molecular weight HA 1.8 MDa (hMwt HA), both alone and in combination with TGFβ1, will enhance human MSC chondrogenesis in an MSC-based tissue engineered construct. This study also evaluated the possibility of supplementing a fibrin:polyurethane scaffold with 1 MDa hyaluronic acid to improve the quality of the fibrin gel-stem cell constructs for the same purpose above. A smaller molecular weight HA was used due to issues relating to handling and viscosity. Both sizes function as high molecular weight HA when considering their biological function.

Thus, our aim is to investigate the effect of hMwt HA supplemented in culture media, or inside the scaffold, on the chondrogenesis of hBM MSCs.

## Materials and Methods

### Poly(Ester-Urethane) Scaffolds Preparation

Poly(ester-urethane) porous sponge (PU) were prepared by using hexamethylene diisocyanate, poly (1-caprolactone) diol and isosorbide diol (1,4: 3,6-dianhydro-D-sorbitol) via a salt leaching-phase inverse technique (Gorna and Gogolewski, 2002). By this procedure interconnected macroporosity ranging from 90 to 300 μm has been uniformly achieved within the sponge. The PU sponge was cut by water-jet (CUTEC AG, Basel, Switzerland) producing cylindrical scaffolds (8 mm diameter × 4 mm height) sterilized in a cold cycle at 37°C via ethylene oxide process and degassed under vacuum for 6 days before to usage.

### Isolation of Human Bone Marrow Derived MSCs

Bone marrow was obtained with full ethical approval (KEK-ZH-NR: 2010–0444/0) and the written consent from patients undergoing routine operations due to bone fracture.

The MSCs were isolated from three different marrow aspirates (two female 1939 and 1992, one male 1958) using Ficoll density separation (Sigma-Aldrich, Buchs, Switzerland) (Table 1).

**TABLE 1:** Legend of the donor details investigated under static conditions.

Donor	Age	Sex
1	79	Female
2	60	Male
3	26	Female

Mononuclear cells were collected from the interphase and the adherent cell fraction was seeded at a density of 50,000 cells/cm^2^ and left to attach for 96 hrs in alpha minimum essential medium (αMEM) (Gibco, Carlsbad, CA, United States), 10% MSC tested fetal bovine serum (FBS) (Pan Biotech, Aidenbach, Germany), 5 ng/ml basic fibroblast growth factor (bFGF) (Peprotech, Rocky Hill, CN, United States) and 1% penicillin/streptomycin (Gibco). When the majority of colonies were confluent, the cells were passaged and seeded into fresh flasks at a cell density of 3,000 cells/cm^2^. The chondrogenic potential of each donor was confirmed using standard techniques.

The hBMSCs isolated from each donor were used separately in three independent experiments.

### Scaffold Seeding and Chondrogenic Differentiation

hMSCs at passage 3 were trypsinized, suspended in a 150 μl fibrinogen-thrombin-solution and evenly seeded at a cell density of 5 × 10^6^ cells/150 μl in cylindrical (8 × 4 mm) macroporous polyurethane (PU) scaffolds. The constructs were fed with four different media for 28 days. Control medium was serum free basal medium containing DMEM high glucose, supplemented with 1% ITS+, 1% Pen/Strep, 1% non-essential amino acid, 50 μg/ml ascorbate-2-phosphate, 5 μM ε-amino-caproic acid (EACA), 10^–7^ M dexamethasone (HA- TGFβ-). This media was further supplemented with 10 ng/mL TGF-β1 (HA- TGFβ+) or with 0.2% 1.8 MDa HA (HA+ TGFβ-) or with both (HA+ TGFβ+). When present, TGF-β1 was only added to the medium. Where mentioned above, the HA groups were supplemented with 1.8 MDa HA (*Stanford Chemicals*) to simulate the synovial fluid concentration under normal conditions (2.3 mg/ml) (Fam et al., 2007). In an additional group, 1 MDa HA (ALB Technology) was added at 0.02% directly into the PU scaffolds (HASc TGFβ+) fed with standard chondrogenic media (HA- TGFβ+).

The culture medium was changed every second day, and conditioned medium was collected for biochemical analysis.

### Gene Expression Analysis: RNA Isolation, cDNA Synthesis, Real Time qPCR

After 7, 14, 21, and 28 days of chondrogenic culture, constructs were harvested, and total RNA was isolated using TRI Reagent (MRC, Cincinnati, OH/Molecular Research Centre Inc.). Total RNA was isolated at day 0 to assess basal gene expression levels.

TaqMan reverse transcription was then performed using 1 μg of total RNA sample, random hexamer primers and TaqMan reverse transcription reagents (Applied Biosystems, Carlsbad, CA, United States).

Real-time PCR was performed using the QuantStudio 6 Flex real-time PCR system (Applied Biosystems). A panel of human genes associated with chondrogenic markers (COL2A1, ACAN, Sox9), the hypertrophic marker COL10A1, osteogenic markers (RunX2; ALP; OC), the Hyaluronan receptor CD44, hyaluronan synthases (HAS1, HAS2, HAS3) and transforming growth factor receptors 1 and 2 (TGFβ-R1 and TGFβ-R2) were investigated.

Primers for RPLP0, COL2A1, COL10A1, ACAN, RunX2, and OC mRNA were synthesized by Microsynth AG (Balgach, Switzerland) (Table 2). Primers for Sox9, ALP, CD44, HAS1, HAS2, HAS3, TGFβ_RI, TGFβ_RII were purchased from Applied Biosystems (Warrington, United Kingdom) (Table 3).

**TABLE 2:** Human oligonucleotide primers and probes used for qRT-PCR.

Gene	Primer forward (5′–3′)	Primer reverse (5′–3′)	Probe (5′ FAM-3′ TAMRA)
COL2A1	5′-GGC AAT AGC AGG TTC ACG TAC A-3′	5′-GAT AAC AGT CTT GCC CCA CTT ACC-3′	5′-CCT GAA GGA TGG CTG CAC GAA ACA TAC-3′
COL10A1	5′-ACG CTG AAC GAT ACC AAA TG-3′	5′-TGC TAT ACC TTT ACT CTT TAT GGT GTA-3′	5′-ACT ACC CAA CAC CAA GAC ACA GTT CTT CAT TCC-3′
ACAN	5′-AGT CCT CAA GCC TCC TGT ACT CA-3′	5′-CGG GAA GTG GCG GTA ACA-3′	5′-CCG GAA TGG AAA CGT GAA TCA GAA TCA ACT-3′
RunX2	5′-AGC AAG GTT CAA CGA TCT GAG AT-3′	5′-TTT GTG AAG ACG GTT ATG GTC AA-3′	5′-TGA AAC TCT TGC CTC GTC CAC TCC G-3′
OC	5′-AAG AGA CCC AGG CGC TAC CT-3′	5′-AAC TCG TCA CAG TCC GGA TTG-3′	5′-ATG GCT GGG AGC CCC AGT CCC-3′
RPLP0	5′-TGG GCA AGA ACA CCA TGA TG-3′	5′-CGG ATA TGA GGC AGC AGT TTC-3′	5′-AGG GCA CCT GGA AAA CAA CCC AGC-3′

*RPLP0, ribosomal protein large P0 housekeeping gene; COL2A1, collagen type 2; COL10A1, collagen type 10; ACAN, aggrecan; RunX2, runt-related transcription factor 2; OC, osteocalcin.*

**TABLE 3:** Assays on demand used for qRT-PCR.

Gene	Assays on demand (ID)
Sox9	Hs00165814_m1
ALP	Hs00758162_m1
CD44	Hs01075861_m1
HAS1	Hs00987418_m1
HAS2	Hs00193435_m1
HAS3	Hs00193436_m1
TGFβ_RI	Hs00610320_m1
TGFβ_RII	Hs00234253_m1

*Sox9, SRY (sex determining region Y)-box 9 cartilage transcription factor; ALP, alkaline phosphatase; CD44, hyaluronan receptor; HAS1, hyaluronan synthase 1; HAS2, hyaluronan synthase 2; HAS3, hyaluronan synthase 3; TGFβ_RI, transforming growth factor β1 Receptor 1; TGFβ_RII, transforming growth factor β1 Receptor 2.*

Relative quantification of target mRNA was determined according to the comparative CT method with hRPLP0 as endogenous control. In addition, the level of gene expression for each gene was determined relative to day 0 monolayer via a ΔΔCT comparison.

### Sulfated Glycosaminoglycans and DNA Quantification

After 28 days of culture, constructs were digested with 1ml proteinase K 0.5 mg/ml at 56°C for 16 h. Total DNA content was measured spectrofluorometrically following reaction with Bisbenzimide Hoechst 33258 dye (Polysciences Inc., Warrington, PA, United States) with purified calf thymus DNA as standard (Lubio Science, Luzern, Switzerland) (Labarca and Paigen, 1980).

Sulfated glycosaminoglycans (GAG) retained within the scaffolds was determined by a direct spectrophotometric microassay according to the dimethylmethylene blue dye method (Sigma-Aldrich, Buchs, Switzerland) at pH 1.5, using bovine chondroitin 4-sulfate sodium salt from bovine trachea (Fluka, St. Louis, MO, United States) (Farndale et al., 1986). Total GAG content of the culture media was also measured to assess the release of matrix molecules from the constructs. All samples containing hyaluronan were blanked with media containing 0.2% hyluronan and DMMB at pH 1.5 was used to eliminate the noisy signal due to the residual interaction between DMMB and Hyaluronan.

### Histology and Staining

After 28 days of culture, the constructs were fixed in 70% methanol and 10 μm specimen sections were cut by cryostat, stained with Safranin O and counterstained with Fast Green to detect proteoglycan presence and proteoglycan-depleted, collagen-rich areas.

### Statistical Analysis

The data were produced from three individual experiments, each carried out with hMSC from a different donor. All experiments were performed in triplicate, quadruplicate or quintuplicates for each group at different timepoints in order to reduce methodological variability. Each measurement was performed in duplicate. Analyses were done between the appropriate control group and treatment groups as well as between different treatment groups, using one way or two ways ANOVA with Tukey’s *Post-hoc* testing whenever required. A significance level of *p* < 0.05 was applied and data are presented as Mean and STDev. Analyses were carried out using the GraphPadPrism 7 software (GraphPad Software Inc., La Jolla, CA, United States).

## Results

### Gene Expression Analysis

**FIGURE 1:**
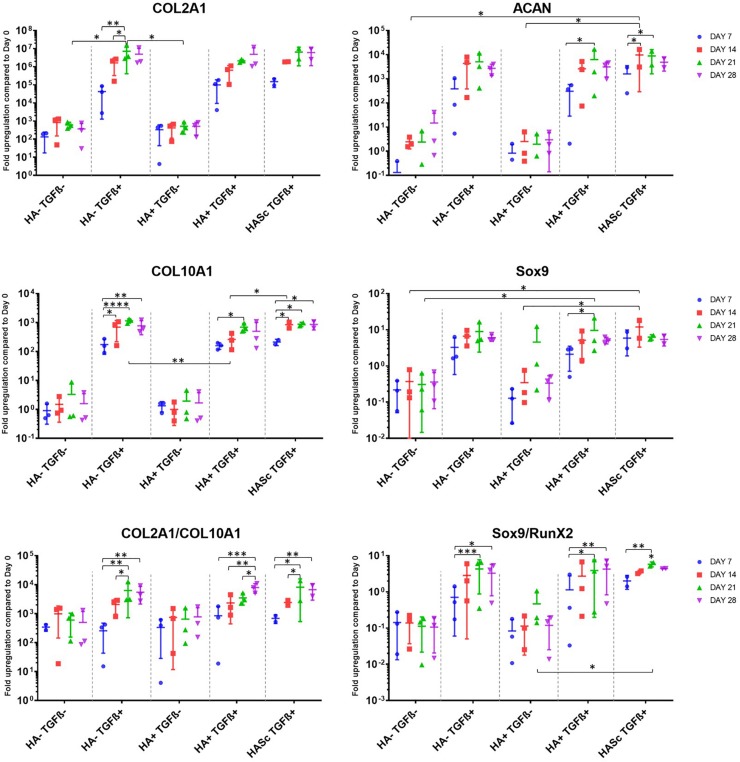
Gene expression measured by qRT-PCR of chondrogenically differentiating hBMSCs-based constructs fed with standard chondropermissive media (HA-TGF-) supplemented with 0.2% sodium hyaluronate (HA+) or without (HA-) and with 10 ng/ml TGFβ1 (TGFβ+) or without (TGFβ-) and harvested at days 7, 14, 21, and 28. Sodium hyaluronate supplemented in media containing TGFβ1 (HA+ TGF+) supports the chondrogenic gene expression of ACAN and Sox9 and reduces the upregulation of the hypertrophic cartilage marker collagen type X. Relative quantification of target mRNA was performed according to the comparative Ct method. Values represent the mean ± SD of three independent hBMSC donors in experimental quadruplicate. Statistical significance was defined as ^∗^*p* < 0.05, ^∗∗^*p* < 0.01, ^∗∗∗^*p* < 0.001, and ^****^p < 0.0001.

A panel of genes associated with chondrogenic differentiation (Collagen type II, Aggrecan, Sox9) were investigated, as well as Collagen type X associated with hMSCs hypertrophic differentiation, genes associated with osteogenic differentiation (RunX2, OC, ALP) and receptors of TGFβ1 (TGFβ_RI, TGFβ_RII) (Figure 1). To gain further understanding of the underlying mechanism, the hyaluronan receptor (CD44) and hyaluronan synthases (HAS1, HAS2, HAS3) were also investigated.

All donors displayed the same trends to a varying degree of magnitude. Among the chondrogenic markers, an overall upregulation of the genes involved in chondrogenic differentiation was observed when the culture media was supplemented with TGFβ1.

All media supplemented with TGFβ showed an overall collagen type II upregulation when compared with TGFβ-free media. The three media formulations containing TGFβ showed a similar profile in terms of expression levels and trend among the different timepoints analyzed. The hypertrophic marker Collagen type X was significantly upregulated from days 7 to 14, 21, and 28 in all the constructs fed with standard chondrogenic media (HA-TGFβ+) with a significant peak at day 21. However, when Hyaluronan is added to the culture media (HA+ TGF+) there is an overall reduction of collagen 10 upregulation at all timepoints. In the second and the last week of chondrogenesis there was no significant increase of collagen 10 compared with day 7. As with HA into the media (HA+ TGFβ+), HA inside the scaffold (HASc TGFβ+) also contributes to reducing the upregulation of the collagen 10 at days 21 and 28 when compared with standard chondrogenic media (HA-TGFβ+). However, the HASc TGF+ group showed a significantly higher Collagen 10 expression from days 7 to 14 compared with HA+ TGF+ group.

All TGFβ supplemented media showed a similar profile with a higher COL2A1/COL10A1 ratio compared with TGFβ depleted media. However, among the TGFβ1 supplemented media, only the group where HA was added (HA+ TGFβ+), showed a significant and progressive increase of the COL2/Col10 ratio from days 7, 14, and 21 to 28. Indeed, for HA depleted media (HA-TGF+), the ratio appears to reach a peak at day 21 although no further significant increase of the ratio was observed from days 21 to 28. When HA was added inside the scaffold (HASc TGF+ group) the COL2A1/COL10A1 ratio reached a peak at day 21 and significantly contributed to an increased ratio from days 7 to 28.

As observed for collagen type II, aggrecan upregulation was observed upon TGFβ supplementation. However, further addition of hyaluronan led to statistically significant increases (Figure 1). Only the groups where hyaluronan was present in the culture media (HA+ TGF+) or added inside the scaffold (HASc TGF+) showed a significant upregulation of aggrecan gene expression from days 7 to 21 in both groups and from day 7 to 14 in the HASc group.

The expression of ACAN at day 14 in HASc TGF+ group was significantly higher compared to the media TGFβ depleted at the same timepoint.

Sox9 expression in the three TGFβ-supplemented media was similar and higher than TGFβ-free media. However, only in groups containing HA do the differences become significant (Figure 1).

In the HASc TGF+ group Sox9 was upregulated earlier at day 14 when it reaches the peak.

The Sox9/Runx2 ratio behaved similarly to the COL2/10 ratio and showed an overall upregulation when the media is supplemented with TGFβ. Particularly, standard chondrogenic media (HA-TGFβ+) showed a significant increase of the ratio from days 7 to 21 and 28. HA supplemented chondrogenic media (HA+ TGF+), also significantly increased the Sox9/RunX2 ratio from days 7 to 21 and 28. In addition, the average value observed in HA+ TGF+ group at day 28, although not significantly different from day 21, showed a tendency to increase compared with day 21, contrary to that seen in the other two TGFβ supplemented groups.

HASc TGF+ group showed a similar profile of HA-TGF+ group with a significant increase of the ratio from days 7 to 21, but not from days 7 to 28.

TGFβRI and II appear to respond in the opposite way to TGFβ supplementation. When the media is TGFβ supplemented, the TGFβRI is more expressed compared with TGF free media groups. Contrarily TGFβRII appear to be slightly less expressed when media is supplemented with TGF compared to TGF free media groups. These observations did not reach significance so the data is not shown.

None of the osteogenic markers (RunX2, ALP, OC), were affected by TGFβ or HA supplementation (data not shown). The expression of HAS 1, 2, and 3 was also largely unaffected by media supplementation (data not shown). CD44 was slightly downregulated over time in all the groups. However, despite a similar trend, no significant downregulation was observed upon media supplementation (data not shown).

### Sulfated Glycosaminoglycan and DNA Quantification

**FIGURE 2:**
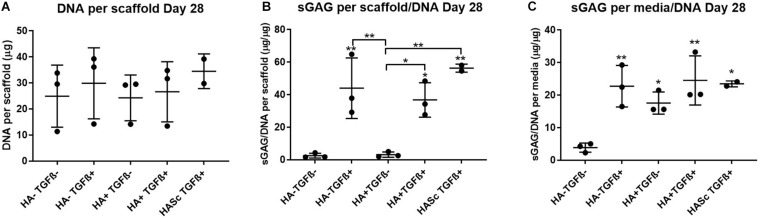
Biochemical analysis of chondrogenically differentiated constructs after 4 weeks in culture shows a significantly higher GAG/DNA when hyaluronic acid is supplemented in culture media (HA+ TGF-). **(A)** Bisbenzimide Höchst 33528 dye was used to quantify the DNA in proteinase K digests of scaffolds. Dimethylmethylene blue (DMMB) at pH 1.5 was used to determine the total amount of sulfated glycosaminoglycan (GAG) produced by mesenchymal stem cells (MSCs). The GAG/DNA ratio was calculated from total DNA and GAG values to show the production of GAG relative to the MSCs present in each group from the proteinase K construct digests **(B)** and the collected culture media **(C)**. All culture media samples containing HA were blanked with media containing HA. Values represent the mean ± SD of three independent hBMSC donors in experimental triplicate or quadruplicate. Statistical significance was defined as *p < 0.05, and **p < 0.01.

After 4 weeks in culture, no significant differences were observed in DNA content among different groups compared with the control (Figure 2). An overall significant increase in sulfated GAG per scaffold/DNA ratio was observed when the medium was supplemented with TGFβ. We also investigated the sGAG and DNA content. After 28 days of chondrogenesis the GAG/DNA ratio per scaffold (Figure 2B) showed a similar GAG deposition in all the media supplemented with TGFβ1, which was significantly higher if compared with the control media (HA-TGF-) and with the hMwt HA supplemented media (HA+ TGF-). However, as it has been shown that part of the sGAG produced within the polyurethane: fibrin scaffold used in this study, are released into the culture media (Wu et al., 2017), the sGAG/DNA ratio per scaffold does not reflect the total amount of sGAG produced by the hMSCs. For this reason, the sGAG/DNA per media was investigated and surprisingly the HA+ TGF- group free from the growth factor TGFβ1, produced significantly higher sGAG/DNA after 28 days of chondrogenesis when compared with the control media HA-TGF- (Figure 2C).

Media GAG showed an overall significant increase in all the experimental groups compared with the control group (HA-TGF-). Particularly, the HA supplemented chondropermissive media TGFβ free (HA+ TGF-), showed a significantly higher media GAG compared with the control group. Also, HA inside the constructs (HASTGF+) were significantly higher than the control.

**FIGURE 3:**
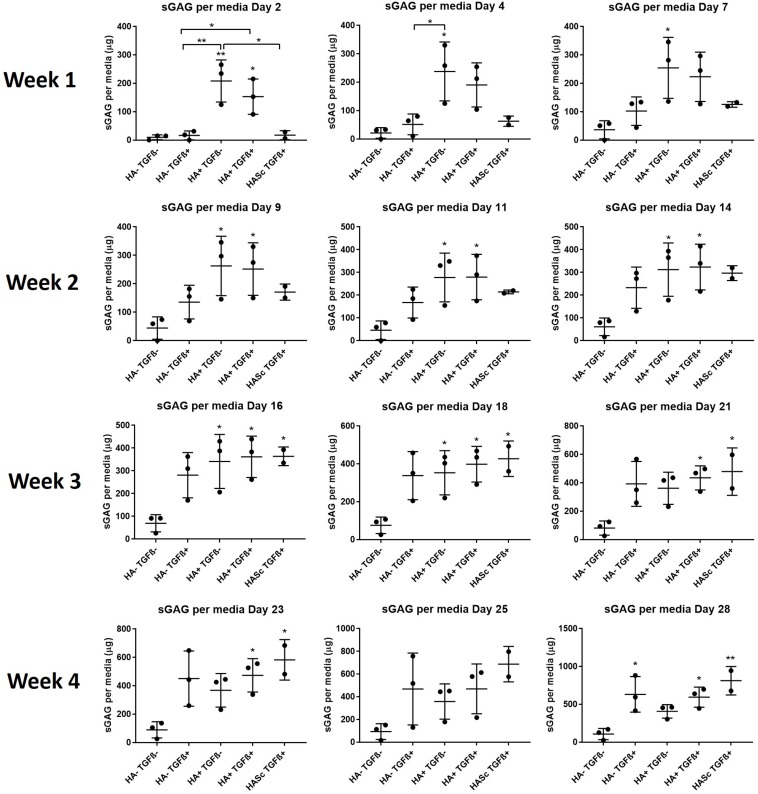
GAG produced and released into culture media from chondrogenically differentiated hBMSC-based constructs over 4 weeks in culture. Chondropermissive medium supplemented with 0.2% sodium hyaluronate (HA+ TGF-) significantly increase sulfated GAG production detected in culture media in the early days of the hBMSC chondrogenesis in the absence of TGFβ. HA supplemented in standard chondrogenic media (HA+ TGF+) did also lead to significant increase in sulfated GAG. sGAG content in culture medium was determined spectrophotometrically following reaction with 1.9-dimethylmethylene blue (DMMB) pH 1.5. All samples containing HA were blanked with medium containing HA. Values represent the mean ± SD of three independent hBMSC donors in experimental quadruplicate or quintuplicate. Statistical significance was defined as *p < 0.05, and **p < 0.01.

**FIGURE 4:**
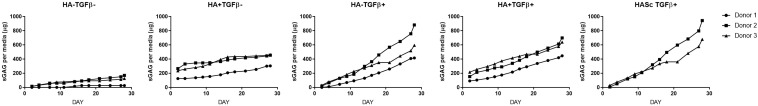
Cumulative sGAG produced and released into culture media from chondrogenically differentiated hBMSC-based constructs along 4 weeks in culture.

With HA containing media (mainly HA+ TGF- and less markedly HA+ TGF+) a significant amount of sGAG was produced by the hMSCs and released into the culture media within the first week (Figures 3, 4). Within the first week of chondrogenesis, the level of GAG in the media supplemented with HA alone (HA+ TGFβ-) was significantly and consistently higher among all three donors when compared with the control TGFβ free medium (HA- TGFβ-) and the standard chondrogenic media containing active TGFβ (HA- TGFβ+) (^∗∗^*p* < 0.01; ^∗^*p* < 0.05) (Figures 3, 4). Particularly, after 2 days of chondrogenic culture, the chondropermissive media supplemented with HA (HA+ TGF-) had significantly higher (*p* < 0.01) GAG release when compared with the standard chondropermissive media (HA-TGFβ-), with the standard chondrogenic media (HA-TGFβ+) (*p* < 0.01) and with the HASTGFβ+ group (*p* < 0.05). The sGAG/DNA of the HA+ TGF- group was comparable with all the other groups fed with TGFβ1 containing media.

At the same timepoint, the GAG level of the standard chondrogenic media HA-supplemented (HA+ TGFβ+) was also significantly higher compared to the control chondropermissive media (HA-TGFβ-) and to the chondrogenic media (HA-TGFβ+).

On day 4, HA+ TGFβ- continued to be significantly higher if compared to HA-TGFβ+ and by day 7, the average value of HA+ TGFβ- continued to be higher than HA-TGFβ+.

However, hyaluronan supplemented chondropermissive media (HA+ TGFβ-) continued to be significantly different from the chondropermissive media control (HA-TGFβ-) until day 18 of chondrogenic culture.

After 14 days of chondrogenesis, the GAG media content of the HA+ TGFβ+ group was greater than the HA+ TGFβ- group and it continued to increase over time until day 28.

By day 16, the GAG released by the HASTGFβ+ group slightly exceeded the average value of all other groups and at the same timepoint became significantly higher than the HA-TGFβ- group, continuing to increase until the end of chondrogenic culture.

### Histology and Safranin O/Fast Green Staining

**FIGURE 5:**
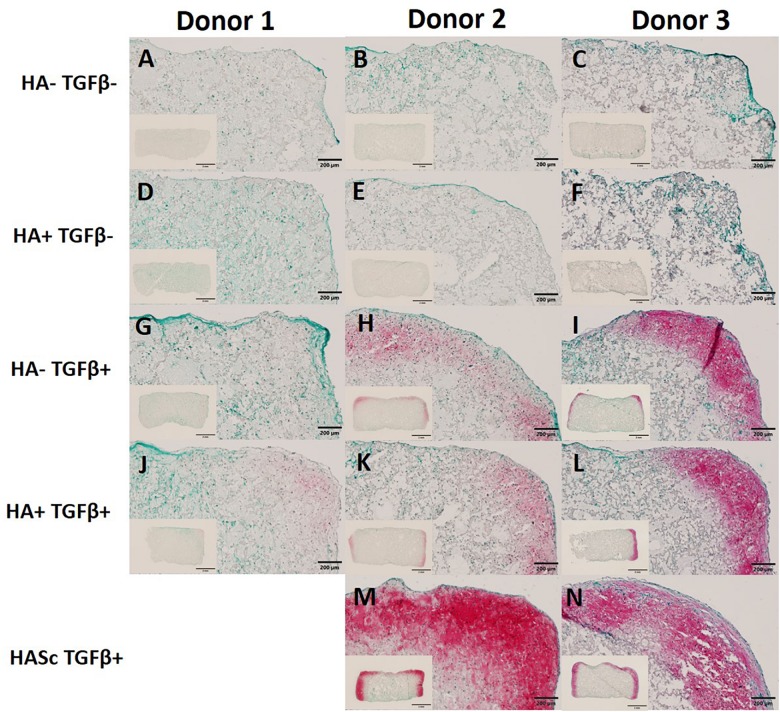
Differentiated constructs stained with safranin O and counterstained with Fast Green after 4 weeks of chondrogenic culture. Images showing higher magnification of the right upper side and the full section of the constructs for three independent hBMSC donors. The HASc TGF+ group shows the best matrix deposition. Scale bar full section: 200 μm, scale bar overview images: 2 mm. Images **(A–C)** show the control group fed with chondropermissive media (HA-TGF–); Images **(D–F)** show the constructs fed with chondropermissive media supplemented with HA (HA+ TGF-); Images **(G–I)** show the constructs fed with chondrogenic media (HA-TGF+); Images **(J–L)** show the constructs fed with chondrogenic media supplemented with HA (HA+ TGF+); Images **(M,N)** show the constructs containing HA and fed with chondrogenic media (HASc TGF+).

In order to show the deposition of sulfated GAGs, after 28 days of chondrogenesis the differentiated constructs were stained with Safranin O and counterstained with Fast green (Figures 5A–N). Positive Safranin O staining was present along the upper and lateral surface of the constructs when the media was supplemented with TGFβ alone (HA-TGF+) and with both factors (HA+ TGF+) with the exception of donor 1 where a slight Safranin O staining was observed only when the media was supplemented with both HA and TGFβ factors (HA+ TGF+).

Stronger positive staining was present in the same region of the constructs when hyaluronan was added directly into fibrin embedding the scaffolds fed with standard chondrogenic media (HASc TGFβ+ group). No staining was observed in the constructs fed with TGFβ1 free media.

## Discussion

Chondrogenic differentiation of mesenchymal stem/stromal cells is routinely evaluated using either monolayer, pellet culture in presence of TGFβ1, or three-dimensional fibrin matrices and insulin-like growth factor-1 (Johnstone et al., 1998; Yoo et al., 1998; Worster et al., 2000, 2001). Thus, most of the *in vitro* culture models to investigate cartilage repair therapies are highly simplified and critical *in vivo* signals that can potentially change the chondrogenic behavior of hBMSCs are lacking.

Particularly relevant *in vivo* functions come from synovial fluid, including acting as a shock absorber and friction reducer, supply of oxygen, nutrients and removal of metabolic waste and CO_2_ from articular chondrocytes (Davies, 1966; Tamer, 2013). Some of these functions are reproduced in *in vitro* models by the cell culture media. However, synovial fluid is known to contain hMwt HA, which to date has been absent from the majority of culture media used in *in vitro* culture systems. This limits the efficacy and reliability of *in vitro* tests, placing a higher burden on *in vivo* models.

**FIGURE 6:**
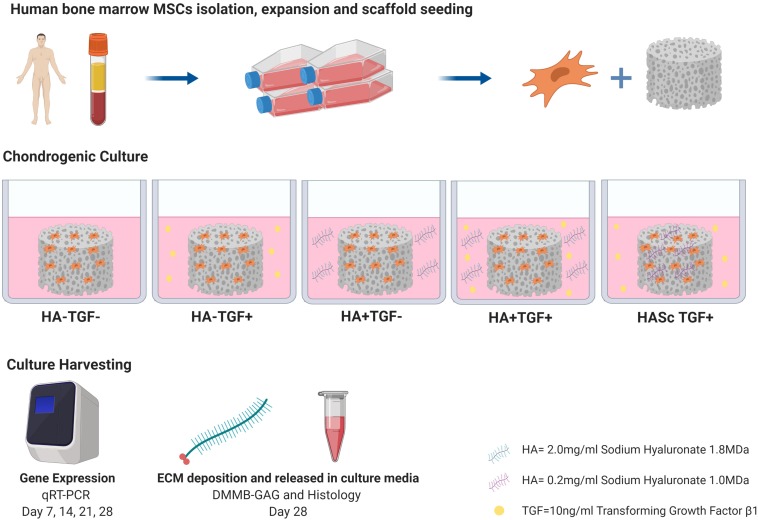
Representative schema of the study experimental steps and design.

To be one step closer to synovial fluid composition, the present study investigated the effect of a chondrogenic media supplemented with a physiological concentration of exogenous hMwtHA, on the differentiation of hBMSCs in a 3D environment (Figure 6). In the present study a pharmaceutical grade HA was used with very low endotoxin contamination (≤0.02 eu/mg or ≤0.05 eu/mg).

Our findings show that monolayer expanded hBMSCs when fed with chondropermissive media supplemented with hMwt HA alone or combined with TGFβ1 undergo, within 28 days, chondrogenic differentiation in 3D microporous polyurethane:fibrin culture system.

Particularly, upon the addition of hMwt HA to chondropermissive medium, we observed: (1) a reduced upregulation of the hypertrophic marker collagen type X, as well as a positive effect on Sox9 and ACAN gene expression level in culture media further supplemented with TGFβ1, (2) a significantly higher sGAG production and release in the culture media within the first week of chondrogenesis and especially in the first 48 h, (3) a more marked extracellular matrix deposition when hMwt HA was supplemented inside the scaffold, (4) consistent and reproducible trends in three different hMSC donors over 28 days of chondrogenic culture.

### hMwt HA Reduces the Expression of Collagen Type X in TGFβ1 Containing Media

Several studies proposed a beneficial effect of hMwt HA in tissue repair following injury or inflammation and the potential role of hyaluronic acid on the differentiation of hMSCs (Shinomura and Kimata, 1990; Jiang et al., 2007; Salamanna et al., 2019). Hypertrophy is one of the main problems related with MSC differentiation in exogenous TGF-β containing media (Johnstone et al., 1998; Goldring et al., 2006). As a result, new protocols to prevent hypertrophy need to be developed to address a stable chondrogenic phenotype, which will help to enable the use of MSCs in clinics. Exogenous hyaluronan, in combination with anti-inflammatory signals, has been shown to act as disease modifying drugs following intra articular delivery with anti-hypertrophic and pro-chondrogenic effects (Prasadam et al., 2013). Similar results were observed when hyaluronic acid was added in a composite 3D collagen gel to address *in vitro* chondrogenesis of human adipose-derived mesenchymal stem cells in co-culture with human chondrocytes. The reduced upregulation of collagen type X appeared to be dose dependent with the best collagen type X reduction at 5% HA (Amann et al., 2017). A reduction of peri-chondrocyte type X collagen was also observed following intraarticular injection of allogeneic MSCs in combination with hyaluronic acid in rabbits (Chiang et al., 2016). The present study confirms the previous findings since the addition of hMwt HA to the chondrogenic media containing TGFβ1 (HA+ TGF+) reproducibly reduced the upregulation of the hypertrophic marker collagen X after 28 days of chondrogenesis. HA inside the scaffold (HASc TGF+) also contributed to reducing the upregulation of collagen type X compared with HA-TGF+, but less markedly than hyaluronic acid supplemented media (HA+ TGF+).

In addition to the reduced upregulation of collagen type X, previous studies have shown an enhanced expression of the chondrogenic markers Sox9 and aggrecan when hMSCs were cultured on hyaluronic acid-coated dishes and the media was supplemented with TGFβ3 (Bhang et al., 2011). The enhanced chondrogenic differentiation of hMSCs due to the interactions with both a specific cell-adhesion matrix as hyaluronic acid and a soluble growth factor as TGFβ3 was shown to be additive (Bhang et al., 2011). The beneficial effect of hyaluronic acid on the upregulation of Sox9 expression was also highlighted in another study conducted by Amann et al. using 1% hyaluronic acid in a 3D collagen hydrogel (Wu et al., 2010; Amann et al., 2017). In the present work we also observed a beneficial effect of the hMwt HA in combination with TGFβ1, on the expression of human chondrogenic markers Sox9 and ACAN. Particularly we observed a statistically significant improvement of the expression level of both genes between days 7 and 21 in media supplemented with both TGFβ1 and hyaluronic acid. HA inside the scaffold significantly upregulated ACAN but did not contribute to a significant upregulation of Sox9 and collagen II, showing a similar profile to the other TGFβ containing media groups.

Upon the addition of hMwt HA, we did not observe a significant upregulation of collagen type II. However, the collagen type II/X mRNA ratio, a chondrocyte differentiation marker, was significantly upregulated over 28 days. A similar upregulation was also present for the HA-TGF+ group but in this case no significant upregulation was observed between days 21 and 28 due to a peak at day 21. This peak was always present for collagen type II, X, and II/X ratio in the HA-TGF+ group. The addition of HA in the culture media appears to attenuate this peak and this attenuation was particularly evident for collagen type X and the II/X ratio. This attenuation could be beneficial for the reduction of unwanted hMSC hypertrophy, which in the standard culture media (HA-TGF+) seems to be particularly pronounced at day 21.

In the present study, CD44 (a cell surface HA receptor; Aruffo et al., 1990) gene expression was unaffected suggesting the possible absence of interaction between exogenous HA and the receptor. This lack of interaction could be due to the high molecular weight of HA used in the present study (1,000 and 1,800 KDa). Several studies reports that low molecular weight fragments (50 KDa) derived from HA degradation bind and activate CD44 receptors during inflammation, promoting cytokine expression (Campo et al., 2009, 2012). However, high molecular weight HA (1,000–7,000 KDa) does not bind CD44 and therefore, does not activate biochemical mediators of damage (Campo et al., 2009; Avenoso et al., 2018). Thus, the lack of upregulation of CD44 indirectly suggests that the hMwt HA did not degrade over time.

### hMwt HA-Supplemented Media Induces a Significant Increase of Early sGAG Synthesis

Chondrocytes embedded in a collagen gel, showed an enhanced sGAG synthesis as well as chondrocyte proliferation after treatment with 0.1 mg/ml 800 KDa HA (Kawasaki et al., 1999).

Akmal et al. (2005) partly confirmed the results of the previous study and showed that HA supplemented culture media in the range between 0.1 and 1 mg/ml enhanced chondrocyte metabolic activity and DNA synthesis, facilitating the deposition of GAG in cartilage tissue between days 9 and 14. Amann et al. (2017) found a significant increase in sGAG production by adipose mesenchymal stem cells seeded in a collagen gel supplemented with different concentrations of HA, with the best GAG/DNA ratio observed with 1% 1.5–1.8 MDa HA. Wu et al. (2017) supplemented the culture media with different concentrations of 1.8 MDa HA and, differently from the previous studies, observed that 2 mg/ml promoted a better preservation of chondrocyte phenotype under joint-kinematic-mimicking treatment especially in terms of collagen type II/I ratio.

None of the studies described above investigated the effect of continuous exposure to hMwt HA supplemented culture media on the chondrogenic differentiation behavior of human bone marrow derived MSCs over 28 days. This could also explain why, in the present work, under static conditions we observed an improvement of the chondrogenic phenotype and an attenuation of the hMSC hypertrophic differentiation following a longer and more constant exposure of the developing constructs to hMwt HA.

In the present work two different types of hyaluronic acid were used: a 1,800 kDa HA was supplemented at 2 mg/ml in the culture media and 1,000 kDa HA was added inside the fibrin gel at 0.2 mg/ml. 1,800 kDa is closer to the size found in synovial fluid, whereas 1,000 kDa was used in the scaffold due to its ease of handling and mixing with the fibrin. Differently from the previous work, in our study we didn’t observe proliferation since the hMSCs DNA content did not increase from days 0 to 28 following the addition of hMwt HA. This might suggest that hMSCs seeded in the polyurethane:fibrin composite were more committed toward chondrogenic differentiation rather than proliferation and hMwt HA did not affect this behavior. It is also necessary to consider that the studies previously described used mainly chondrocytes and not MSCs. Thus, we can assume that the commitment level of the cell type affects the response to hyaluronic acid and based on previous studies, chondrocytes are more inclined to proliferation than MSCs which, in our study, appeared to be more prone to differentiation with the proper stimuli.

The reasons for the early GAG production and release into the media are unclear but previous studies have shown that hyaluronic acid promoted a chondroprotective effect following intra articular injection *in vivo*, with an increase of the endogenous proteoglycan and glycosaminoglycan synthesis and a suppression of the degradation (Kobayashi et al., 2004; Altman et al., 2015). HA promoted the mobilization of the newly synthesized proteoglycan from the pericellular matrix to the interterritorial cartilage matrix, providing protection against degradation and contributing to the strengthening of the interterritorial cartilage matrix (Kikuchi et al., 2001; Altman et al., 2015). This mobilization could have happened also in our system but due to the early sGAG production, we could hypothesize that there is insufficient pericellular ECM to be able to retain the newly produced ECM causing its release into the media.

The early GAG production might also be explained by another interesting phenomena: macromolecular crowding. Macromolecular crowding (MMC), a biophysical phenomenon known to accelerate *in vitro* biological processes, accelerate and increases ECM deposition (Minton, 2001; Kumar et al., 2015; Shendi et al., 2019). The crowded environment is achieved by supplementation of the culture media with a synthetic or natural macromolecule (Kumar et al., 2015). Thanks to the excluded volume effect exerted by the macromolecule, the culture media better recapitulates the crowded physiological *in vivo* environment and this is the reason of the *in vitro* acceleration of biological processes as ECM deposition (Kumar et al., 2015; Prewitz et al., 2015). Previous studies suggested that MMC can enhance GAG deposition during hMSC differentiation (Welter et al., 2009; Prewitz et al., 2015) and have shown culture media supplemented with 0.2% 1.8 MDa hyaluronic acid acts as macromolecular crowding agent (Zhou et al., 2008; Shendi et al., 2019). Therefore, the early sGAG production observed within the first week of chondrogenesis and particularly within the first 48 h of culture, could be explained by a potential macromolecular crowding effect exerted by hyaluronic acid supplemented in culture media. However, we did not observe an increased expression of collagen type I, II, III, or aggrecan.

### hMwt HA in Fibrin Gel Enhances the ECM Deposition

Histological analysis of ECM deposition provided evidence of positive Safranin O staining when the media was supplemented with TGFβ1. Addition of HA in the culture media slightly improved the ECM deposition. However, when HA was added inside the scaffold a stronger ECM deposition was observed. Simple mixing of HA to a fibrin composite as performed here would be a clinically applicable carrier gel that could be used to deliver MSCs locally into a cartilage defect. Our results indicate this would be beneficial when considering matrix deposition.

In a previous study it was observed that HA inside a hydrogel might affect cell behavior as it reduces oxygen concentration (Amann et al., 2017). Several studies suggest that low oxygen is favorable for chondrocyte phenotype maintenance and could be used as stimulus for hMSC chondrogenic differentiation (Buckley et al., 2010; Sheehy et al., 2012).

Exogenous hyaluronic acid injected into rabbit knee joints was incorporated into articular cartilage (Antonas et al., 1973). This incorporation could happen also over time in our system when the differentiating hMSCs are in contact with exogenous HA supplemented directly in the fibrin gel or in the culture media. HA supplemented in media or in the fibrin gel might facilitate the accumulation of extracellular matrix since HA is one of the main components, thus explaining the better extracellular matrix deposition in HASc TGF+ group.

In addition, the gene expression results of Sox9, ACAN and collagen type 10, the sGAG deposition and the histological analysis, suggests that the location of hyaluronic acid might play a role on the chondrogenic response of hMSCs seeded inside a 3D scaffold.

Despite mesenchymal stem cells holding great promise for the treatment of orthopedic injury and disease, donor variation during chondrogenic differentiation has severely hindered their clinical use (Kim et al., 2018). Donor variation makes interpretation of the results more difficult and often, when statistical analysis is performed on several donors, some useful results can be hidden. In these cases, it is better to aim at personalized medicine and to study the behavior of individual donors.

In the present study, despite the small donor variation observed, the trends of gene expression analysis, sGAG production and deposition, and histological analysis was consistent, reliable and reproducible among three different human mesenchymal stem cell donors.

## Clinical Significance and Potential Applications

*In vitro* models aim to recapitulate the complexity of *in vivo* systems. Most of the work on chondrogenic differentiation of hMSCs use a culture media with a very simple composition which is an oversimplification of the *in vivo* system. In order to mimic more closely the *in vivo* physiology, current models need to be improved to reflect the complexity of the synovial joint environment and to try to more accurately predict *in vivo* outcomes. In the current climate of animal welfare and “3Rs,” more accurate *in vitro* models will be crucial to prevent *in vitro* artifacts and to enable more accurate prescreening of potential cartilage repair therapies. Producing reliable results *in vitro* that are more representative of the *in vivo* outcome, will also help to reduce the need for animal studies. Therefore, in addition to the standard approaches that aim to engineer cartilage tissue, it is worth to pursue the bioengineering of a more complex culture media. In addition, the association of HA media with human mesenchymal stem cells and fibrin gel, which is currently used in clinical practice, makes the whole system more relevant for clinics. HA supplemented media, being one step closer to the synovial fluid, can be used to make preclinical testing systems more predictable of clinics for several applications:

(1) to screen and evaluate potential cartilage and osteochondral repair therapies which involves the use of human MSCs (differentiating cartilage) or human chondrocytes (mature cartilage) in *in vitro* models or *ex vivo* systems simulating traumatic defects or diseases,

(2) to screen new biomaterials designed for tissue engineering and clinical application purposes,
(3) to screen new drug candidates for cartilage regeneration and osteochondral repair,
(4) to study the behavior of autologous or allogeneic human stem cell for intra articular joint injection in 2D/3D *in vitro* environments,
(5) to potentially increase the clinical effectiveness of tissue engineered constructs developed in a more synovial fluid-like environment.

## Conclusion and Perspectives

Overall, our results demonstrate that the constant supplementation of 1.8 MDa hMwt HA at 2 mg/ml to the culture media or the addition of 1 MDa hMwt HA into the scaffolds at 0.2 mg/ml, had a positive effect on the intrinsic capacity of hMSCs to produce ECM. Increased sGAG production occurred especially in the early days of chondrogenesis when the hMwt HA was supplemented in culture media; while a better matrix deposition was observed when hMwt HA was added in the scaffold. Considering all the evidence mentioned above, further studies are ongoing to investigate the composition of the ECM released into the culture media and deposited inside the constructs.

First it would be interesting to understand the underlying mechanism by which the addition of HA into the media leads to an increased early production and why this effect decreases with time in culture. Finding these answers would allow a better understanding how to prevent the release of the sGAG and this would help to rapidly establish an initial ECM pericellular matrix. This would function as an improved baseline to assist in the effective deposition of further ECM that might contribute to a final improved/stronger ECM quality and quantity.

Eventually, physicochemical culture media factors that affect the cellular response and matrix deposition, such as viscosity (Laurent and Ogston, 1963; Laurent, 1995), osmolality (Laurent and Ogston, 1963; Urban et al., 1993; Palmer et al., 2001), diffusion properties (Hansen et al., 2016), and the potential interaction between HA and other molecules in the culture media (Kuznetsova et al., 2015), should be further investigated.

At the gene expression level, exogenous hMwt HA reduced the upregulation of the hypertrophic cartilage marker collagen type X. Therefore, a more stable hMSC chondrogenic differentiation can also be achieved through the development of a more physiological synovial-like bioengineered culture media. The bioengineered media might also reproduce *in vitro* another important function exerted by hMwt HA *in vivo*, namely its function as a shock absorber and friction reducer during dynamic loading. It is an ideal lubricant in the synovial joints due to its shear-dependent viscosity (Laurent et al., 1996; Morgese et al., 2018). Sliding motion and dynamic compression induces hMSCs chondrogenesis (Schatti et al., 2011) and joint-kinematic-mimicking mechanical loading combined with HA media improves matrix production in a bovine chondrocyte cartilage engineered model (Wu et al., 2017). In addition, specific stimuli mimicking human articular joint kinematics may promote the development of a functional articular surface–synovial interface (Grad et al., 2005). For these reasons it will be of high interest to perform additional studies in this direction to further increase the complexity by including mechanical stimuli typically experienced within articular joints. This will be possible thanks to our joint-simulating bioreactor that mimics the knee joint kinematics (Wimmer et al., 2004; Schatti et al., 2011). Thus, future work will include long-term loading experiments over several weeks to investigate if the lubricating capability of HA supplemented media can act synergistically with joint kinematic motion to address the chondrogenic differentiation behavior of hMSCs. This study will be useful on experimental level to improve our understanding of the homeostasis mechanisms of articulating joints and on a clinical level might serve as baseline to improve knee joint rehabilitation protocols after trauma or disease.

## Data Availability Statement

All relevant datasets generated for this study are included in the article.

## Ethics Statement

The studies involving human participants were reviewed and approved by the Cantonal Ethics Commission, University of Zurich, Zurich (Ethics Commission reference number KEK ZH Nr 2010-0444/0). The patients provided their written informed consent to participate in this study.

## Author Contributions

GM designed and performed the experiments, analyzed the data, and wrote the manuscript. GM and MS reviewed and edited the manuscript. MS and MA worked on funding acquisition. MS, MA, and AE supervised the whole work. All authors reviewed the results and approved the final version of the manuscript.

## Conflict of Interest

The authors declare that the research was conducted in the absence of any commercial or financial relationships that could be construed as a potential conflict of interest.
